# Mott variable-range hopping transport in a MoS_2_ nanoflake†

**DOI:** 10.1039/c9ra03150b

**Published:** 2019-06-06

**Authors:** Jianhong Xue, Shaoyun Huang, Ji-Yin Wang, H. Q. Xu

**Affiliations:** Beijing Key Laboratory of Quantum Devices, Key Laboratory for the Physics and Chemistry of Nanodevices, Department of Electronics, Peking University Beijing 100871 China hqxu@pku.edu.cn syhuang@pku.edu.cn; Beijing Academy of Quantum Information Sciences West Bld. #3, No. 10 Xibeiwang East Rd., Haidian District Beijing 100193 China; Beijing Academy of Quantum Information Sciences Beijing 100193 China; NanoLund, Division of Solid State Physics, Lund University Box 118 S-221 00 Lund Sweden

## Abstract

The transport characteristics of a disordered, multilayered MoS_2_ nanoflake in the insulator regime are studied by electrical and magnetotransport measurements. The MoS_2_ nanoflake is exfoliated from a bulk MoS_2_ crystal and the conductance *G* and magnetoresistance are measured in a four-probe setup over a wide range of temperatures. At high temperatures, we observe that ln *G* exhibits a −*T*^−1^ temperature dependence and the transport in the nanoflake dominantly arises from thermal activation. At low temperatures, where the transport in the nanoflake dominantly takes place *via* variable-range hopping (VRH) processes, we observe that ln *G* exhibits a −*T*^−1/3^ temperature dependence, an evidence for the two-dimensional (2D) Mott VRH transport. Furthermore, we observe that the measured low-field magnetoresistance of the nanoflake in the insulator regime exhibits a quadratic magnetic field dependence ∼ *αB*^2^ with *α* ∼ *T*^−1^, fully consistent with the 2D Mott VRH transport in the nanoflake.

## Abstract 2

The transport characteristics of a disordered, multilayered MoS_2_ nanoflake in the insulator regime are studied by electrical and magnetotransport measurements.

## Graphical abstract

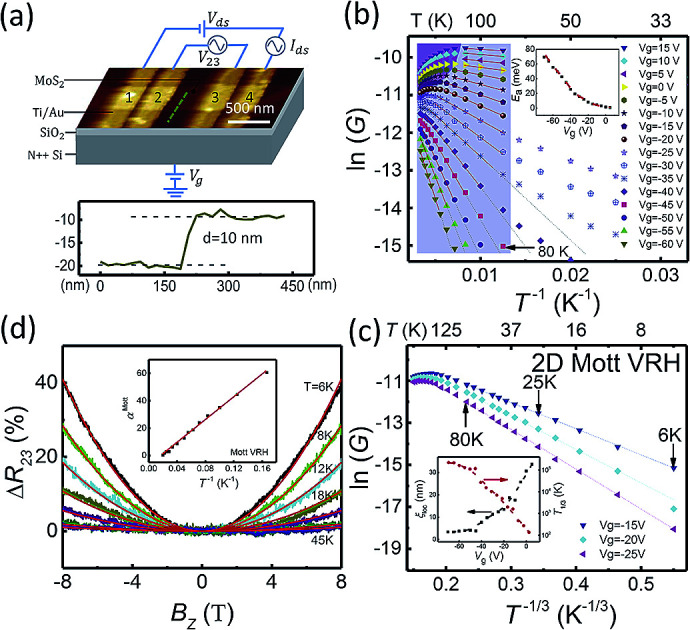

Despite the rapid developments in MoS_2_ layered materials and device applications, the nature of charge transport still remains elusive, since the experimentally measured carrier mobility in the materials is significantly lower than theoretical prediction.^1–4^ Disorders, such as sulphur vacancies, charge impurities, surface absorbents, charged traps at MoS_2_ layer–substrate interfaces,^5–8^*etc.*, strongly influence the transport properties of the layered MoS_2_ materials. Generally, in a disordered system,^9^ transport can be divided into two different regimes, separated by the mobility edge *E*_C_. Tuning the Fermi energy *E*_F_ across the mobility edge causes a metal–insulator transition^10–12^ from extended to localized states or *vice versa*. In the insulating regime, where carriers are all frozen to localized states at energies below the mobility edge *E*_C_ at zero temperature, carrier transport can take place *via* thermal activation at relatively high temperatures and *via* variable-range hopping (VRH) at low but finite temperatures. When the carrier transport is predominantly due to thermal activation, the conductance (*G*) exhibits a temperature (*T*) dependence of ln *G* ∼ −*T*^−1^ and, thus, the characteristics activation energy *E*_a_ can be extracted from the Arrhenius plot of the measured conductance *G* as a function of temperature *T*. When temperature goes higher than a critical value (∼100 K in many compound semiconductor materials), phonon scattering can become significantly strong, leading to a characteristic temperature dependence that the conductance decreases with increasing temperature. At low temperatures, the current is carried *via* VRH processes in which a localized electron at the Fermi level moves to another localized state in an optimum hopping distance. The optimum distance is determined by the tradeoff between the lowest energy differences and the shortest hopping distances. In a noninteracting *d*-dimensional system, the density of states *N*_F_ at the Fermi level is finite and the conductance can be well described by the Mott VRH mechanism^13^ with ln *G* ∼ −*T*^−*p*^ where exponent *p* = 1/(*d* + 1). Pollak,^14,15^ Efros and Shklovskii^16^ pointed out that a soft gap can be opened up at the Fermi level by taking into account the long range electron–electron Coulomb interactions. The density of states at the Fermi level thus vanishes and the conductance should be described by the so called ES VRH mechanism^17–19^ with exponent *p* = 1/2 in ln *G* ∼ −*T*^−*p*^, independent of the dimension.

Due to the two-dimensional (2D) nature and the natural presence of disorders, layered MoS_2_ offers a renewed platform to investigate VRH mechanisms of 2D transport. Thermally activated transport,^20^ and nearest neighbor hopping (NNH) and VRH transport^5–8,21–24^ have been observed and discussed in many recent experimental studies of disorders, layered MoS_2_. Wu *et al.*,^5^ Ghatak *et al.*^7^ and Jariwala *et al.*^8^ studied the transport properties of atomically thin layer MoS_2_ devices and found that the transport in the MoS_2_ layers are well described by the 2D Mott VRH mechanism over a wide range of temperatures *T* (300–30 K). Qiu *et al.*^6^ examined the transport properties of few-layer MoS_2_ devices at low carrier densities and found that 2D Mott VRH mechanism dominate only the transport in the MoS_2_ layer in a low *T* region (100–20 K), while in the high *T* region (300–100 K) NNH dominates the transport in the layers. Liang *et al.*^24^ found a similar transition between NNH and VRH while the corresponding transition temperature is 70 K. Lo *et al.*^21^ studied transport in a monolayer MoS_2_ nanoflake and found that both the Mott and the ES VRH models can provide satisfactory explanation for their measurements at *T* < 190 K. Very recently, Kim *et al.*^22^ and Papadopoulos *et al.*^23^ studied transport in few-layered nanoflakes of *n*-butyllithium treated polymorphic MoS_2_. While Kim *et al.*^22^ found that their transport measurements are described by the 2D Mott VRH mechanism, Papadopoulos *et al.*^23^ showed contradictorily that the transport in their few-layered MoS_2_ nanoflakes is well described by the ES VRH mechanism. In all the above mentioned works, the temperature-dependent measurements of the conductance or the resistance are exclusively analyzed to determine transport mechanisms. Beside some controversies are present in assignment of VRH transport mechanisms, no carrier density dependent transition between different transport mechanisms was reported in these works.

Magnetotransport measurements could also be used to distinguish the two VRH mechanisms in the disordered MoS_2_ layers. In a common situation where the magnetoresistance shows a quadratic dependence on magnetic field as *αB*^2^, the coefficient *α* has different temperature dependences for the two VRH transport mechanisms.^25^ In the Mott VRH regime the coefficient *α* ∝ *T*^−3/(*d*+1)^, whereas in the ES VRH regime the coefficient *α* ∝ *T*^−3/2^. Thus, magnetotransport measurements could be analyzed together with the temperature and carrier density dependent measurements to identify the transport mechanisms in disordered MoS_2_ nanoflakes. We notice that although there are some works^26^ reporting the magnetoresistance of MoS_2_, no efforts to distinguish between the Mott and ES VRH transports based on magnetotransport measurements has been made yet.

In this work, we report on an experimental study of the transport characteristics of a disordered MoS_2_ nanoflake by electrical and magnetotransport measurements in a four-probe setup over a temperature range of 6 to 300 K and at different carrier densities. The study is focused on the insulator regime where the Fermi energy *E*_F_ lies below the mobility edge *E*_C_. When *E*_F_ is tuned close to *E*_C_, the characteristics of thermally activated transport and phonon scattering are observed in the conductance measurements of the nanoflake. When *E*_F_ is tuned far below *E*_C_, the measured ln *G* shows a −*T*^−1^ dependence at relatively high temperatures and a −*T*^−1/3^ dependence at relatively low temperatures. A good quadratics magnetic field dependence ∼ *αB*^2^ of the magnetoresistance is also observed in the nanoflake and a *T*^−1^ dependence of coefficient *α* is extracted. These electrical and magnetotransport measurements provide a solid evidence that the Mott VRH rather than ES VRH transport is the dominant transport mechanism in our disordered MoS_2_ nanoflake in the insulating regime at low temperatures.

The MoS_2_ nanoflakes studied in this work are obtained by exfoliation from a commercially available bulk MoS_2_ crystal. The exfoliated MoS_2_ nanoflakes are transferred onto a highly doped silicon substrate covered with a 300 nm-thick layer of SiO_2_ on top. Electrical contacts are prepared using electron-beam lithography (EBL) for pattern definition, electron-beam evaporation for deposition of 5 nm-thick titanium and 50 nm-thick gold, and lift-off process. Fig. 1(a) shows an atomic force microscope (AFM) image of a fabricated device measured for this work and the schematic for the measurement circuit setup. The MoS_2_ nanoflake in the device has a width of *W* ∼ 400 nm and a thickness of *t* ∼ 10 nm, see the AFM line scan measurements across an edge of the nanoflake shown in the lower panel of Fig. 1(a). The device consists of four metal stripe contacts, which we labeled as contacts 1 to 4 as in Fig. 1(a). These contacts have a width of 200 nm. The edge-to-edge distances between contacts 1 and 2, between contacts 2 and 3, and between contacts 3 and 4 are 80, 450 and 100 nm, respectively. The measurements are performed in a Physical Property Measurement System (PPMS) cryostat, which provides temperatures in a range of 300 to 2 K and magnetic fields up to 9 Tesla. The four-probe setup is adopted in the measurements, in order to exclude the contact resistances, in which a source–drain bias voltage *V*_ds_ is applied between contacts 1 and 4, the channel current *I*_ds_ and the voltage drop (*V*_23_) between contacts 2 and 3 are simultaneously recorded, see the circuit setup in Fig. 1(a). The Fermi level *E*_F_ in the nanoflake is modulated by a voltage *V*_g_ applied to the silicon substrate (back gate).

**Fig. 1:**
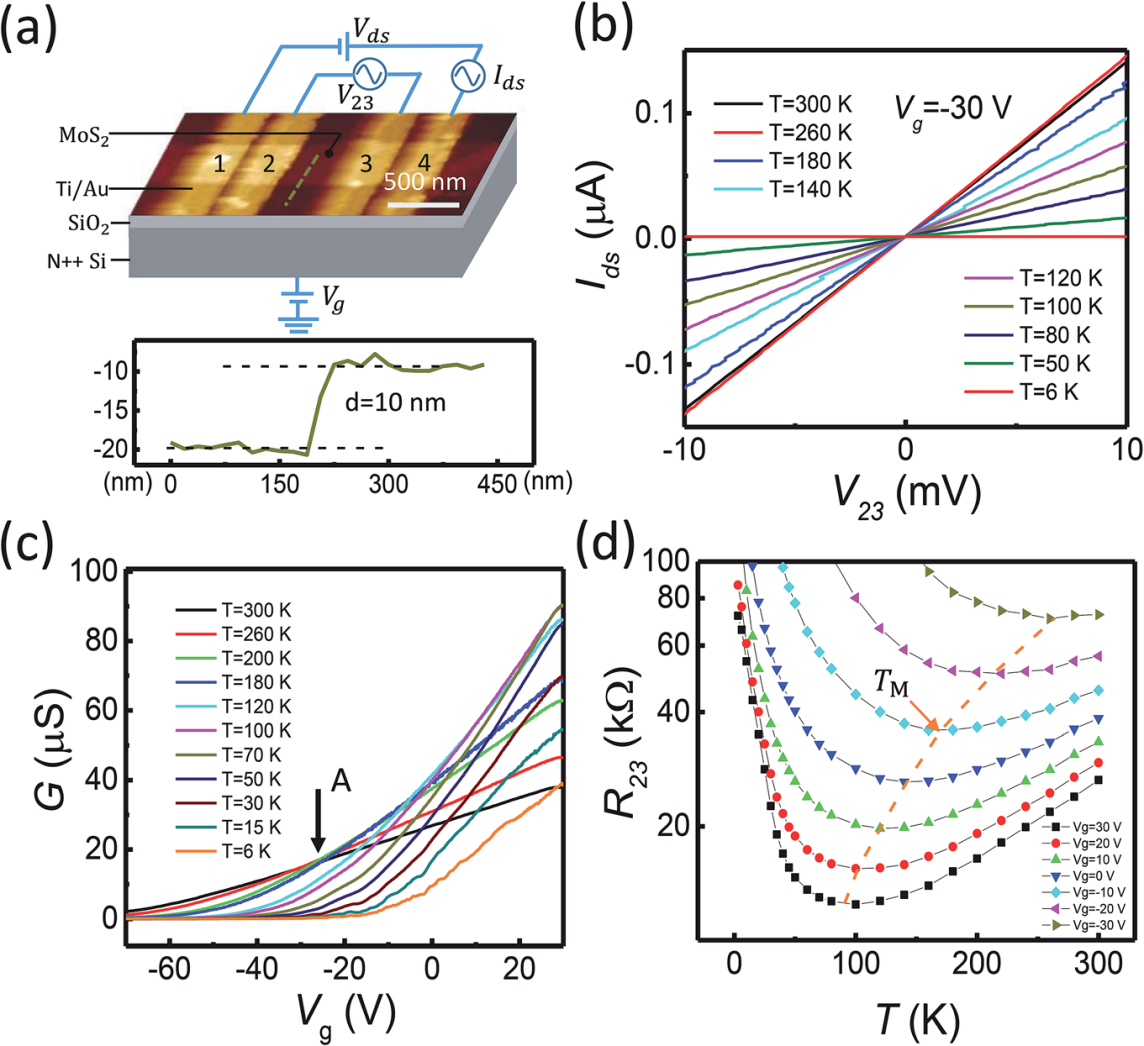
(a) AFM image of the MoS_2_ nanoflake device and schematic view of the device layer structure and measurement setup (top panel), and height profile measured using AFM along the dashed line in the AFM image (bottom panel). Here it is shown that the MoS_2_ nanoflake in the device has a thickness of 10 nm. (b) Source–drain current *I*_ds_*vs.* voltage *V*_23_ measured for the device at temperatures from 6 to 300 K and at back gate voltage *V*_g_ = −30 V. (c) Transfer characteristics of the field-effect device at temperatures from 6 to 300 K. (d) Resistance of the nanoflake plotted against temperature *T* at different back gate voltages. *T*_M_ marks the temperature position at which the resistance has a minimum in the curve measured at each back gate voltage. The dashed line connecting the values of *T*_M_ at different back gate voltages is the guide to the eyes.

Fig. 1(b) shows the measured channel current *I*_ds_ as a function of the voltage drop *V*_23_ between contacts 2 and 3 for the device shown in Fig. 1(a) at a fixed back gate voltage of *V*_g_ = −30 V and different temperatures. It is seen in the figure that the measured *I*_ds_–*V*_23_ curves are straight lines. The good linearity is found in all the measured *I*_ds_–*V*_23_ curves over a wide range of back gate voltages and of temperatures, which ensures that the transport characteristics of the MoS_2_ channel are extracted from the measurements. Fig. 1(c) shows the measured channel conductance *G* = *I*_ds_/*V*_23_ as a function of back gate voltage *V*_g_ at different temperatures. It is seen that the device is a typical n-type transistor. The channel conductance shows very different temperature dependence at high back gate voltages (on the right side of point A) and at low back gate voltages (on the left side of point A), where the crossover point A is located at *V*_g_ ∼ −30 V, as indicated by a black arrow in Fig. 1(c). At the high back gate voltages (on the right side of point A), the conductance *G* is increased with decreasing temperature and then becomes decreased with further lowering temperature. At the low back gate voltages (on the left side of point A), however, the conductance *G* is monotonously decreased with decreasing temperature in the entire measured temperature range (from 300 to 6 K). The observed temperature dependence of the conductance at low temperatures indicates that the MoS_2_ channel is in the insulating regime and the Fermi energy *E*_F_ lies below the mobility edge *E*_C_ throughout the entire measurement range of back gate voltages. The characteristic conductance increase with decreasing temperature observed at high back gate voltages and high temperatures arises from the interplay between the thermal activation transport and phonon scattering. This interplay phenomena could be better visualized by plotting the resistance *R*_23_ as a function of temperature measured at different back gate voltages as shown in Fig. 1(d). Here, we can clearly recognize that at a given high back gate voltage *V*_g_ > −30 V, there exists a characteristic temperature *T*_M_, at which the resistance has a minimum. Apparently, *T*_M_ increases with decreasing back gate voltage, see the yellow dashed line in Fig. 1(d), and can reach a temperature as low as ∼100 K at *V*_g_ = 30 V. Physically, at such a high back gate voltage, the Fermi level *E*_F_ is close to the mobility edge *E*_C_ and a significant number of carriers can be excited to the extended states located at energies above the mobility edge at high temperatures. Thus, at *T* > *T*_M_, the observed fact that the resistance decreases with decreasing temperature is mainly due to reduction in phonon scattering with decreasing temperature. However, at *T* < *T*_M_, the phonon scattering becomes less important and the resistance becomes closely related to the number of carriers which are thermally excited to the extended states. As the back gate voltage *V*_g_ decreases, the Fermi level *E*_F_ is gradually moved away from the mobility edge *E*_C_, leading to an increase in *T*_M_ as seen in Fig. 1(d).

Fig. 2 shows the Arrhenius plot of the measured conductance as a function of temperature at different back gate voltages. In the temperature region of 80 K < *T* < *T*_M_, *i.e.*, the shaded part except for the upper-left corner region in the figure, relatively large thermal kinetic energy assisted transport dominates and the temperature dependence of the conductance can be well modelled by the thermally activated transport^20^ as *G*_a_ = *G*_0_e^−*E*_a_/*k*_B_*T*^. Here, *G*_0_ is the conductance at the high temperature limit, *E*_a_ = (*E*_C_ − *E*_F_) is the activation energy, and *k*_B_ is the Boltzmann constant. The extracted activation energy *E*_a_ is shown in the inset of Fig. 2 as a function of the back gate voltage. The activation energy *E*_a_ decreases linearly with increasing *V*_g_ from −70 to −30 V and turns to saturate with further increasing *V*_g_ to the positive side, in good agreement with the fact that the Fermi level moves closer to the mobility edge with increasing back gate voltage.

**Fig. 2:**
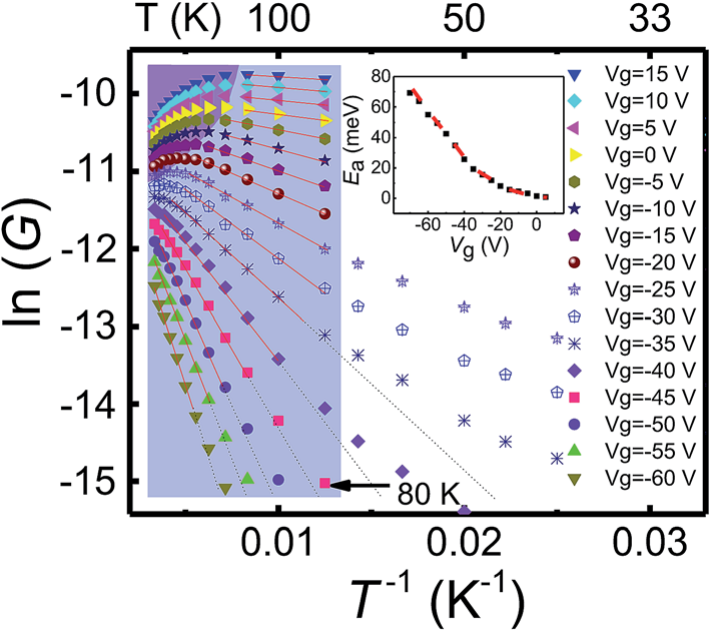
ln *G* plotted against *T*^−1^ (inverse of temperature) for the device at different back gate voltages. The region on the left marked by grey color is for the measurements at temperatures higher than ∼80 K, at which the transport in the nanoflake is well described by the thermal activation mechanism. The upper-left corner marked by light purple color is the region of the measurements at high temperatures and high positive gate voltages, where the characteristics of phonon scattering in the layered MoS_2_ is observed. Lines in the grey colored region are straight line fits to the measured data. The inset shows the extracted activation energy *E*_a_ from the straight line fits in the grey color region as a function of back gate voltage *V*_g_. The red dashed line in the inset is a guide to the eyes to clarify the change of *E*_a_.

However, the thermally activated transport model does not describe the measurements in the low temperature region [the right, unshaded part of Fig. 2] as seen from the deviations from the fitting lines in the region. Physically, in this low temperature region, VRH conduction becomes dominant and is responsible for the temperature dependent characteristics of the measured conductance. In theory, the conductance in VRH mechanisms can be described as1
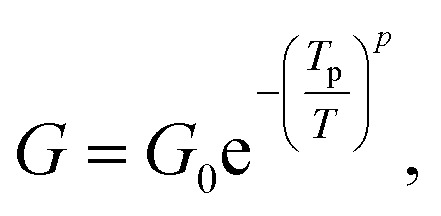
where *T*_p_ is the characteristic temperature and the exponent *p* depends on VRH mechanism. For the Mott VRH conduction, the exponent *p* is dimension dependent and has a value of *p* = 1/3 in a 2D system. For the ES VRH conduction, the exponent is dimension independent and has a value of *p* = 1/2. To identify whether the Mott VRH mechanism or the ES VRH mechanism play a dominant role in determining the transport characteristics of the MoS_2_ nanoflake, we plot the measured conductance as a function of *T*^−1/2^ and of *T*^−1/3^ in Fig. 3(a) and (b), respectively. It is seen in Fig. 3(a) that the measured data cannot be fitted by straight lines, indicating that the ES VRH mechanism does not describe the transport behavior of the nanoflake in this low temperature range. However, in Fig. 3(b), straight-line fits agree excellently with the measured data over the entire temperature range of 6 to 80 K. Thus, the transport in the MoS_2_ nanoflake in this temperature range is most likely governed by the 2D Mott VRH process. We have also checked and fitted our data against the 3D Mott VRH model (see ESI†), in which the exponent takes a value of *p* = 1/4, and found large deviations, indicating that the transport in the nanoflake should be of the 2D nature. The 2D nature of the transport in our nanoflake is also supported by the angular dependent magnetoresistance measurements as shown in ESI.†

**Fig. 3:**
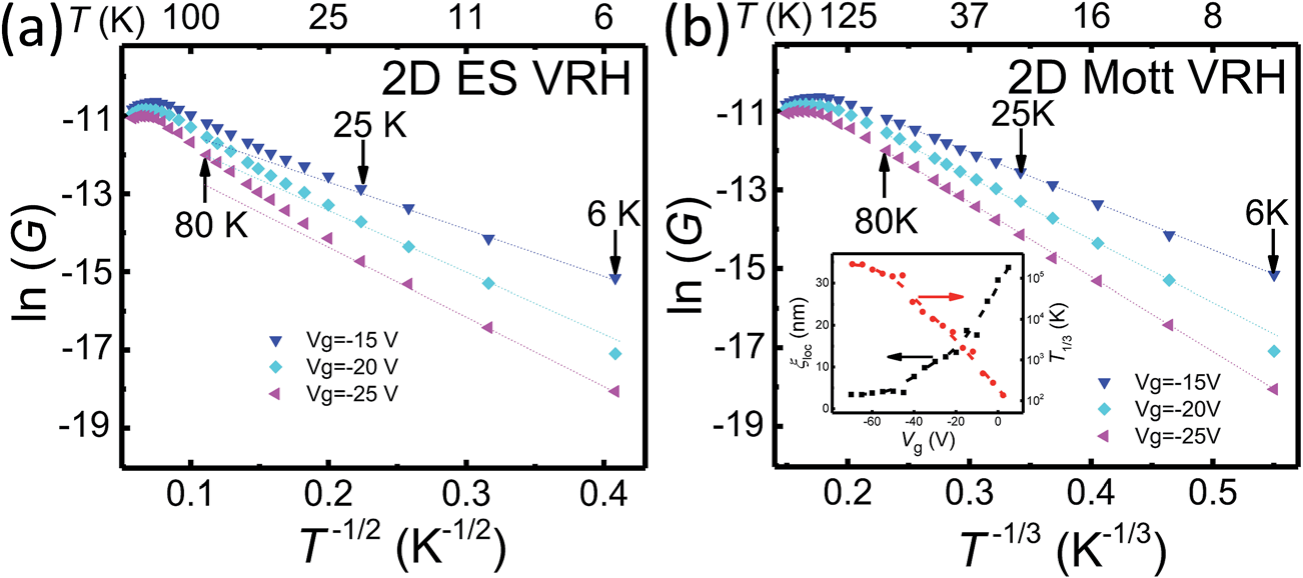
(a) ln *G* plotted against *T*^−1/2^ for the device measured at different back gate voltages. Lines are straight line (2D ES VRH theory) fits to the measurement data at low temperatures. Clearly, the straight line fits do not describe the measurement data at temperatures of 25 to 80 K. (b) The same as in (a) but plotted against *T*^−1/3^. Lines are straight line (2D Mott VRH theory) fits to the measurement data at temperatures of 6 to 80 K. Here, excellent fits are obtained over this range of temperatures. The inset shows the extracted characteristic temperature *T*_1/3_ and localization length *ξ*_loc_ as a function of back gate voltage *V*_g_ for the MoS_2_ nanoflake based on the 2D Mott VRH theory.

Employing the 2D Mott VRH mechanism, we can further analyze the temperature dependence of the conductance to extract the characteristic temperature *T*_1/3_ and localization length *ξ*_loc_ in the nanoflake at different gate voltages *V*_g_. The results are shown in the inset of Fig. 3(b). It is seen that *T*_1/3_ monotonically decreases from ∼10^5^ to ∼10^2^ K with increasing *V*_g_ from −70 to 5 V. Theoretically, *T*_1/3_ is related to the localization length as *T*_1/3_ = 13.8/(*k*_B_*N*_F_*ξ*_loc_^2^), where *N*_F_ is the density of states at Fermi level. The *N*_F_ can be determined from the gate voltage dependence of the activation energy *E*_a_ by taking into account the quantum capacitance *C*_d_ as^12,27^2
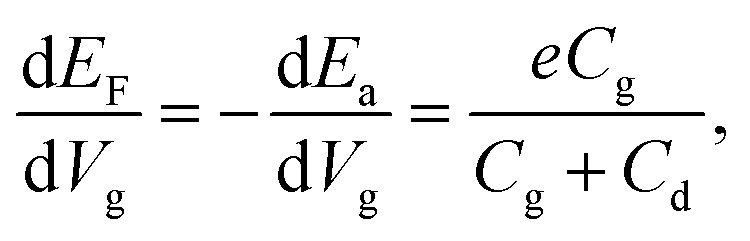
with *C*_d_ = *e*^2^*N*_F_. Here, *C*_g_ is the unit area capacitance and can be obtained from 
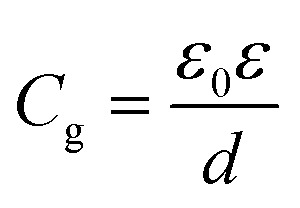
 with *ε*_0_ being the vacuum permittivity, *ε* and *d* being the dielectric constant and layer thickness of SiO_2_. The calculated values of *C*_d_ and *N*_F_ are from 6 to 10 μF cm^−2^ and from 3.75 to 6.25 × 10^13^ eV^−1^ cm^−2^, respectively. Thus, the derived localization length of the nanoflake is found to increase from 3.4 to 33.7 nm with increasing *V*_g_ from −70 to 5 V, as shown in the inset of Fig. 3(b).

Fig. 4(a) show the magnetoresistance characteristics at different temperatures in the 2D VRH transport regime at *V*_g_ = −20 V with the magnetic field *B* applied perpendicular to the MoS_2_ nanoflake. Here, the MoS_2_ nanoflake is in the *x*–*y* plane, the current flow is along the *x* axial direction, and the magnetoresistance is defined as Δ*R*_23_ = [*R*(*B*) − *R*(*B* = 0)]/*R*(*B* = 0). Clearly, the magnetoresistance shows a positive quadratic dependence on magnetic field *B* at temperatures below ∼50 K. In the wave-function shrinkage model,^28–31^ the positive quadratic magnetoresistance is attributed to the contraction of the electronic wave function at traps in a magnetic field, thus leading to a reduction of hopping probability. Quantitatively, in the wave-function shrinkage model, the magnetoresistance in the Mott VRH regime can be expressed as^25^3
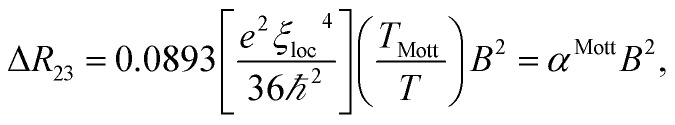
where *T*_Mott_ is a characteristic temperature, *ℏ* is the reduced Planck constant and the prefactor *α*^Mott^ is proportional to *T*^−1^. Note that in the ES VRH regime, the magnetoresistance can be found by replacing (*T*_Mott_/*T*) in eqn (3) with (*T*_ES_/*T*)^3/2^. Thus, the magnetoresistance also shows a positive quadratic magnetic field dependence, but the prefactor *α*^ES^ is proportional to *T*^−3/2^ instead of *T*^−1^. We fit the positive quadratic magnetoresistance curves at different temperatures in Fig. 4(a) based on the wave-function shrinkage models in both the Mott VRH and the ES VRH regime. The derived prefactors *α*^Mott^ and *α*^ES^ are shown in Fig. 4(b). Clearly, the extracted *α*^ES^ as a function of *T*^−3/2^ shown in the inset of Fig. 4(b) exhibits a significant deviation, while the extracted *α*^Mott^ displays an excellent agreement with the *T*^−1^ dependence. Thus, the 2D Mott VRH mechanism rather than the ES VRH mechanism is identified for the electron transport in the MoS_2_ nanoflake at low temperatures, in good agreement with the analysis shown in Fig. 3.

**Fig. 4:**
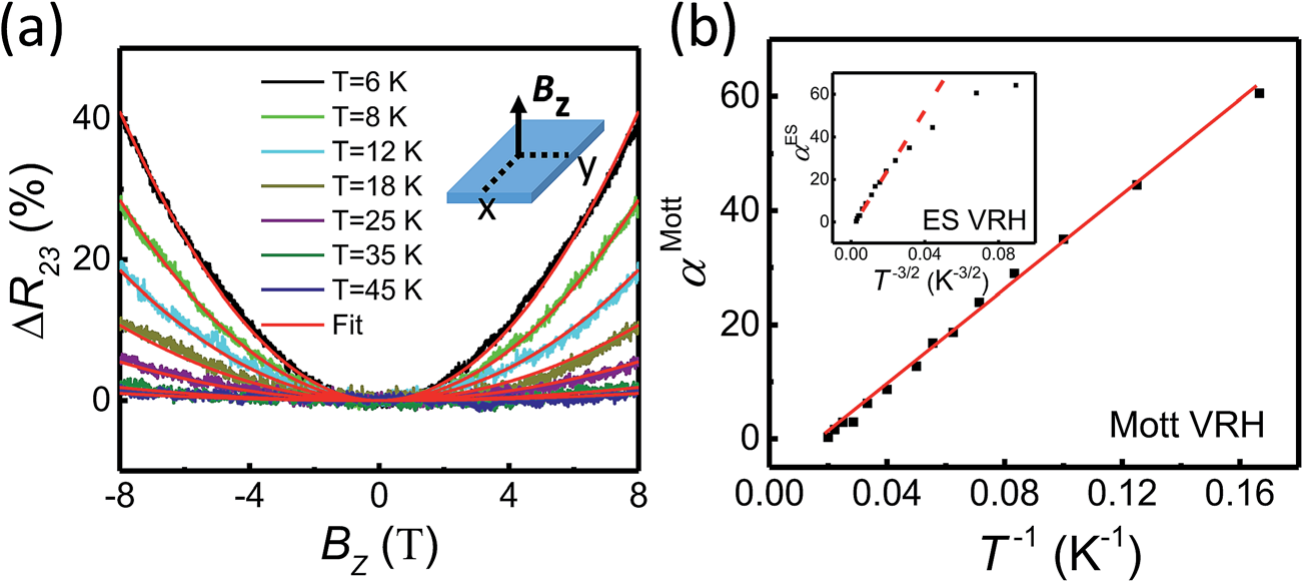
(a) Magnetoresistance as a function of magnetic field *B*_*z*_ applied perpendicular to the MoS_2_ nanoflake plane (as shown in the inset) measured at back gate voltage *V*_g_ = −20 V and at different temperatures. Red solid lines are fits to the measured data based on the wave-function shrinkage model. (b) Prefactor *α*^Mott^ as a function of *T*^−1^ extracted from the measured magnetoresistance curves at different temperatures *T*. The red solid line presents the predicted values of *α*^Mott^ by the wave-function shrinkage model in the Mott VRH regime. The inset shows the prefactor *α*^ES^ extracted from the same magnetoresistance measurements as a function of *T*^−3/2^. The dashed line in the inset shows the results that would be predicted by the wave-function shrinkage model in the ES VRH regime.

In conclusion, the transport characteristics of a disordered MoS_2_ nanoflake have been investigated in details over a wide range of temperatures in the insulator regime, where the Fermi level *E*_F_ in the nanoflake is tuned with use of the back gate voltage to lie below the mobility edge *E*_C_. At relatively high temperatures, the nanoflake exhibits activation transport characteristics. The activation energy *E*_a_ = *E*_C_ − *E*_F_, which measures the energy distance between the mobility edge *E*_C_ and the Fermi energy *E*_F_, is extracted in the nanoflake. It is found that the activation energy *E*_a_ decreases with increasing back gate voltage at low back gate voltages and turn to saturate towards zero at high back gate voltages. At sufficiently low temperatures, the transport characteristics of the nanoflake are found to be governed by VRH processes. To identify whether the Mott or the ES VRH mechanism plays a dominant role in the system at this low temperature region, the temperature dependent conductance and magnetoresistance have been measured and analyzed. It is found that in this low temperature region the ln *G* shows a −*T*^−1/3^ temperature dependence and the prefactor in the quadratic magnetic field dependent magnetoresistance scales with temperature as *T*^−1^. These results provide exclusive evidences that the 2D Mott VRH transport is the dominant transport mechanism at low temperatures in the insulating regime of our disordered MoS_2_ nanoflake.

## Conflicts of interest

There are no conflicts of interest to declare.

## Supplementary Material

RA-009-C9RA03150B-s001

## References

[cit1] Radisavljevic B., Radenovic A., Brivio J., Giacometti V., Kis A. (2011). Nat. Nanotechnol..

[cit2] Wang Q. H., Kalantar-Zadeh K., Kis A., Coleman J. N., Strano M. S. (2012). Nat. Nanotechnol..

[cit3] Yu Z., Ong Z.-Y., Li S., Xu J.-B., Zhang G., Zhang Y.-W., Shi Y., Wang X. (2017). Adv. Funct. Mater..

[cit4] Kaasbjerg K., Thygesen K. S., Jacobsen K. W. (2012). Phys. Rev. B: Condens. Matter Mater. Phys..

[cit5] Wu J., Schmidt H., Amara K. K., Xu X., Eda G., Ozyilmaz B. (2014). Nano Lett..

[cit6] Qiu H., Xu T., Wang Z., Ren W., Nan H., Ni Z., Chen Q., Yuan S., Miao F., Song F., Long G., Shi Y., Sun L., Wang J., Wang X. (2013). Nat. Commun..

[cit7] Ghatak S., Pal A. N., Ghosh A. (2011). ACS Nano.

[cit8] Jariwala D., Sangwan V. K., Late D. J., Johns J. E., Dravid V. P., Marks T. J., Lauhon L. J., Hersam M. C. (2013). Appl. Phys. Lett..

[cit9] Mott N., Pepper M., Pollitt S., Wallis R. H., Adkins C. J. (1975). Proc. R. Soc. London, Ser. A.

[cit10] Radisavljevic B., Kis A. (2013). Nat. Mater..

[cit11] Baugher B. W., Churchill H. O., Yang Y., Jarillo-Herrero P. (2013). Nano Lett..

[cit12] Ovchinnikov D., Allain A., Huang Y. S., Dumcenco D., Kis A. (2014). ACS Nano.

[cit13] Mott N. F. (1968). J. Non-Cryst. Solids.

[cit14] Pollak M. (1971). Discuss. Faraday Soc..

[cit15] Knotek M. L., Pollak M. (1972). J. Non-Cryst. Solids.

[cit16] Efros A. L., Shklovskii B. I. (1975). J. Phys. C: Solid State Phys..

[cit17] Khondaker S. I., Shlimak I. S., Nicholls J. T., Pepper M., Ritchie D. A. (1999). Solid State Commun..

[cit18] Zhang Y., Dai P., Levy M., Sarachik M. P. (1990). Phys. Rev. Lett..

[cit19] Aharony A., Zhang Y., Sarachik M. P. (1992). Phys. Rev. Lett..

[cit20] Ayari A., Cobas E., Ogundadegbe O., Fuhrer M. S. (2007). J. Appl. Phys..

[cit21] Lo S. T., Klochan O., Liu C. H., Wang W. H., Hamilton A. R., Liang C. T. (2014). Nanotechnology.

[cit22] Kim J. S., Kim J., Zhao J., Kim S., Lee J. H., Jin Y., Choi H., Moon B. H., Bae J. J., Lee Y. H., Lim S. C. (2016). ACS Nano.

[cit23] Papadopoulos N., Steele G. A., van der Zant H. S. J. (2017). Phys. Rev. B.

[cit24] Liang S., Yang H., Renucci P., Tao B., Laczkowski P., Mc-Murtry S., Wang G., Marie X., George J. M., Petit-Watelot S., Djeffal A., Mangin S., Jaffres H., Lu Y. (2017). Electrical spin injection and detection in molybdenum disulfide multilayer channel. Nat. Commun..

[cit25] Su T.-I., Wang C.-R., Lin S.-T., Rosenbaum R. (2002). Phys. Rev. B: Condens. Matter Mater. Phys..

[cit26] Park T.-E., Suh J., Seo D., Park J., Lin D.-Y., Huang Y.-S., Choi H.-J., Wu J., Jang C., Chang J. (2015). Appl. Phys. Lett..

[cit27] Zhu W., Low T., Lee Y.-H., Wang H., Farmer D. B., Kong J., Xia F., Avouris P. (2014). Nat. Commun..

[cit28] Bloom F. L., Wagemans W., Kemerink M., Koopmans B. (2007). Phys. Rev. Lett..

[cit29] Zhou Y.-B., Han B.-H., Liao Z.-M., Wu H.-C., Yu D.-P. (2011). Appl. Phys. Lett..

[cit30] Sadu R., Haldolaarachchige N., Chen D., Young D. P. (2013). Appl. Phys. Lett..

[cit31] Zhang Y., Ning H., Li Y., Liu Y., Wang J. (2016). Appl. Phys. Lett..

