# Supplementary material for: Mott variable-range hopping transport in a MoS2 nanoflake
Source: RSC Adv. 2019 Jun 6;9(31):17885–90. doi: 10.1039/c9ra03150b (PMC9064683; doi:10.1039/c9ra03150b)
Supplement: RA-009-C9RA03150B-s001 [file RA-009-C9RA03150B-s001.pdf]

Electronic Supplementary Material (ESI) for RSC Advances.
This journal is © The Royal Society of Chemistry 2019

Supplemental Materials for

Mott variable-range hopping transport in a MoS_2 nanoflake

Jianhong Xue,[1] Shaoyun Huang,[1,*] Ji-Yin Wang,[1] and H. Q. Xu[1, 2,3,+]

[1]*Beijing Key Laboratory of Quantum Devices, Key Laboratory for the Physics and Chemistry of Nanodevices and Department of Electronics, Peking University, Beijing 100871, China*

[2]*Beijing Academy of Quantum Information Sciences, West Bld. #3, No.10 Xibeiwang East Rd., Haidian District, Beijing 100193, China*

[3]*NanoLund and Division of Solid State Physics, Lund University, Box 118, S-221 00 Lund, Sweden*

Correspondence should be addressed to: +Professor H. Q. Xu (hqxu@pku.edu.cn) and *Dr. Shaoyun Huang (syhuang@pku.edu.cn)

1. Temperature dependence of the electron mobility in the MoS_2 nanoflake

Figure 1(c) of the main article shows the measured channel conductance $G{=}I_{ds}/V_{23}$ of the studied MoS_2 nanoflake device as a function of back gate voltage V_g at different temperatures, see Figure 1(a) in the main article for the device structure and the measurement setup. The electron mobility of the nanoflake at different temperatures can be extracted from the measured conductance curves shown in Figure 1(c) of the main article using the following equation

$$\mu = \frac{L_{23}}{W \times C_g} \times \frac{\mathrm{d}G}{\mathrm{d}V_g}, \tag{1}$$

where the channel length L_{23} is 650 nm and the channel width W is 400 nm. C_g is the unit area capacitance, which can be estimated using $C_g = \frac{\varepsilon_0 \varepsilon}{d}$, where ε_0 is the vacuum permittivity, $\varepsilon = 3.9$ is the dielectric constant of SiO_2, d = 300 nm is the thickness of SiO_2. Figure 1 in this Supporting Information shows the extracted mobility from the measured conductance shown in Figure 1(c) of the main article. The results shown in Figure 1 manifest distinctly different temperature dependences at low and at high temperatures. In the low temperature region (the left side of the figure), the mobility decreases with decreasing temperature, which is consistent with the fact that the nanoflake is in the insulating regime at all the gate voltages considered in this work. In the high temperature region (the right side of the figure), the mobility decreases with increasing temperature, showing the characteristic influence of phonon scattering on the electron transport in the nanoflake. As discussed in the main article, in this high temperature region, the transport is predominantly carried out by the carriers which are thermally excited

to the extended states located above the mobility edge. However, at these high temperatures, phonons become active and phonon scattering plays a dominant role in limiting carrier transport, resulting in a characteristic dependence of the carrier mobility on temperature, $\mu \propto T^{-\gamma}$, where constant γ depends on the specific phonon scatting mechanisms. The fitting at the high temperature side of Figure 1(a) gives a value of $\gamma \approx 1.74$, which is much close to the theoretically predicted value for MoS_2 optical phonon scattering in monolayer MoS_2 ($\gamma \approx 1.52$)[1] and is very different from that in bulk crystals ($\gamma \approx 2.6$)[2]. The experimental results are consistent with the fact that the thickness of the MoS_2 nanoflake is 10 nm in the device.

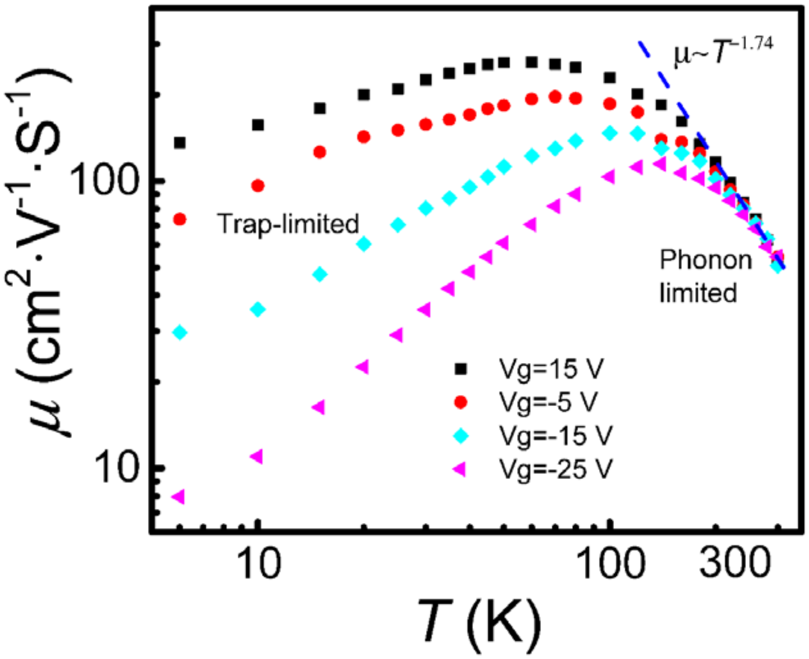

Figure 1. Electron mobility μ extracted for the MoS_2 nanoflake studied in the main article as a function of temperature at different gate voltages. At low temperatures, μ decreases with decreasing temperature, showing that the nanoflake is in the insulating regime at the gate voltages considered. At high temperatures, μ decreases with increasing temperature as $\mu \propto T^{-1.74}$, implying that the carrier transport is dominantly limited by optical phonon scattering in the nanoflake.

2. Fitting our measurement data to the 3D Mott VRH model

In the main article, we show that the measured conductance at low temperatures of 6 K < T < 80 K are fitted excellently by the 2D Mott variable-range hopping (VRH) model, but cannot be well described by the 2D ES VRH model. Here we show in Figure 2 that the measurement data could not be fitted by the 3D Mott VRH model. In the 3D Mott VRH model, the $ln\, G$ is

related to temperature as $-T^{-1/4}$. In Figure 2, we replot the measurements data (in $ln\,G$) as a function of $T^{-1/4}$. It is clearly seen that a straight line fit in the high temperature region from 80 to 25 K does not extended to the low temperature region from 25 to 6 K well. Thus, the 3D Mott VRH model fails to describe the transport behavior in the nanoflake in the full temperature range from 80 to 6 K.

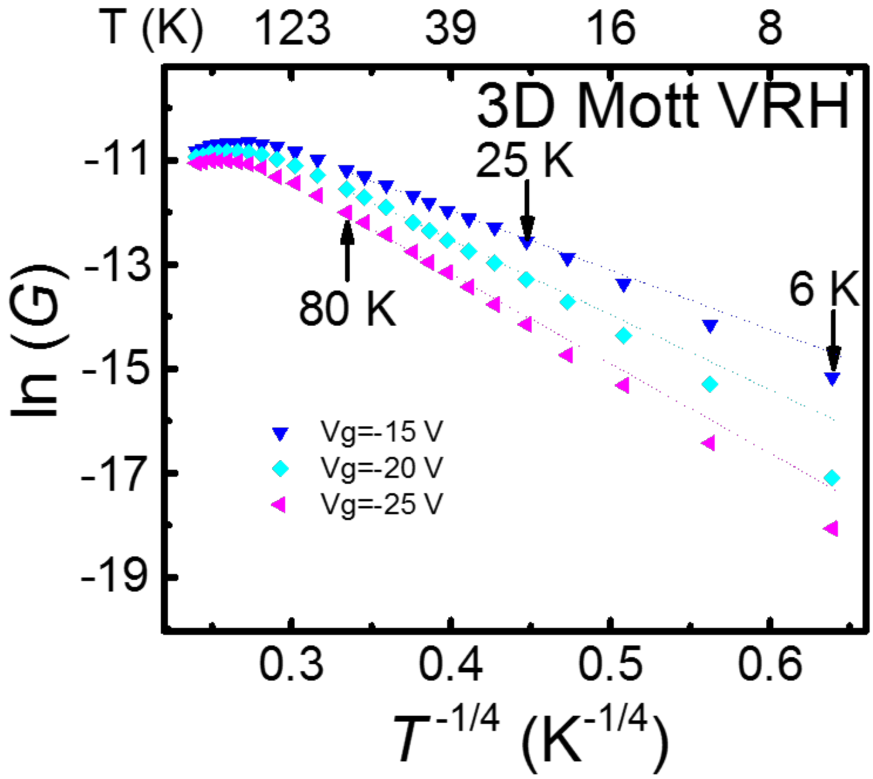

Figure 2. $ln\,G$ plotted against $T^{-1/4}$ at three representative back gate voltages. Clearly, the data in the temperature range of 6 to 80 K could not be fitted using single straight lines.

3. Magnetoresistance measurements with the magnetic fields applied at different angles

To show further that the transport in the MoS_2 nanoflake is predominantly of 2D nature, we measure the magnetoresistance of the nanoflake device with the magnetic fields applied at different angles θ. The results of the measurements for the device at temperature T=6K and back gate voltage V_g=−20 V are shown in Figure 3(a). Here, as shown in the inset of Figure 3(a), the nanoflake is in the *x*-*y* plane, the transport takes place along the *x* direction, and the magnetic fields are applied in the *y*-*z* plane, i.e., always perpendicular to the current direction. The magnetoresistance is defined as $\Delta R_{23} = [R(B) - R(B=0)]/R(B=0)$, where $R = V_{23}/I_{ds}$, see Figure 1(a) of the main article for the measurement circuit setup. At $\theta = 90^{\circ}$, the magnetoresistance shows a positive quadratic magnetoresistance as we discussed in the main article. At $\theta = 0^{\circ}$, a weak negative magnetoresistance is observed. This weak negative

magnetoresistance arises from the finite thickness (~10 nm) of the nanoflake and thus the suppression of back scattering by the top and bottom surfaces of the nanoflake by the in-plane magnetic field. However, the thickness of the nanoflake is very small, the negative magnetoresistance is small in magnitude, e.g., it is less than 3% even at the in-plane magnetic field of 8 T. Figure 3(b) shows the normalized magnetoresistance, $\Delta R_{23}^{+} = \Delta R_{23} - \Delta R_{23}^{\theta=0^{\circ}} \cdot \cos(\theta)$, as a function of perpendicular component of the magnetic field $B_z = B \cdot \sin\theta$. Here, the $\Delta R_{23}^{\theta=0^{\circ}}$ is the measured magnetoresistance at in-plane magnetic fields and can be acquired from Figure 3(a) at $\theta = 0^{\circ}$. It is seen that the normalized magnetoresistance is solely dependent on the perpendicular component of the applied magnetic field. Overall, the results shown in this supplementary note imply that the transport in the nanoflake is predominantly of the 2D nature, but a small 3D transport characteristic could be present which may cause some small but observable deviations from the predictions of 2D transport theory.

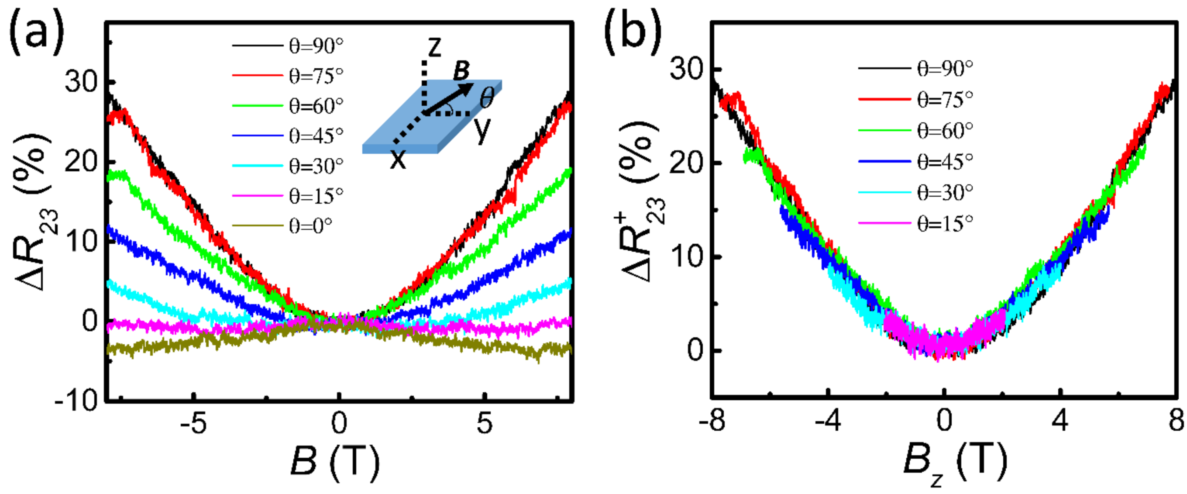

Figure 3. (a) Magnetoresistance measured for the nanoflake device with the magnetic fields applied at different orientations θ as shown in the inset at temperature T= 6 K and at back gate voltage V_g= −20 V. A positive quadratic magnetoresistance is observed at $\theta = 90^{\circ}$ and a very weak magnetoresistance is observable at $\theta = 0^{\circ}$. (b) Normalized magnetoresistance plotted against the perpendicular component of the applied magnetic field B_z. Here, the normalized magnetoresistance is obtained from (a) by subtracting the in-plane contributions from the measured magnetoresistance values.

References

[1] K. Kaasbjerg, K. S. Thygesen, and K. W. Jacobsen, Phys. Rev. B **85**, 115317 (2012).

[2] R. Fivaz and E. Mooser, Phys. Rev. **163**, 743 (1967).
